# Tissue Specificity of Human Angiotensin I-Converting Enzyme

**DOI:** 10.1371/journal.pone.0143455

**Published:** 2015-11-23

**Authors:** Olga V. Kryukova, Victoria E. Tikhomirova, Elena Z. Golukhova, Valery V. Evdokimov, Gavreel F. Kalantarov, Ilya N. Trakht, David E. Schwartz, Randal O. Dull, Alexander V. Gusakov, Igor V. Uporov, Olga A. Kost, Sergei M. Danilov

**Affiliations:** 1 Chemical Faculty, M.V.Lomonosov Moscow State University, Moscow, Russia; 2 Bakulev Center for Cardiovascular Surgery, Moscow, Russia; 3 Institute of Urology, Moscow, Russia; 4 Department of Medicine, Columbia University, New York, NY, United States of America; 5 Department of Anesthesiology, University of Illinois at Chicago, Chicago, IL, United States of America; Max-Delbrück Center for Molecular Medicine (MDC), GERMANY

## Abstract

**Background:**

Angiotensin-converting enzyme (ACE), which metabolizes many peptides and plays a key role in blood pressure regulation and vascular remodeling, as well as in reproductive functions, is expressed as a type-1 membrane glycoprotein on the surface of endothelial and epithelial cells. ACE also presents as a soluble form in biological fluids, among which seminal fluid being the richest in ACE content - 50-fold more than that in blood.

**Methods/Principal Findings:**

We performed conformational fingerprinting of lung and seminal fluid ACEs using a set of monoclonal antibodies (mAbs) to 17 epitopes of human ACE and determined the effects of potential ACE-binding partners on mAbs binding to these two different ACEs. Patterns of mAbs binding to ACEs from lung and from seminal fluid dramatically differed, which reflects difference in the local conformations of these ACEs, likely due to different patterns of ACE glycosylation in the lung endothelial cells and epithelial cells of epididymis/prostate (source of seminal fluid ACE), confirmed by mass-spectrometry of ACEs tryptic digests.

**Conclusions:**

Dramatic differences in the local conformations of seminal fluid and lung ACEs, as well as the effects of ACE-binding partners on mAbs binding to these ACEs, suggest different regulation of ACE functions and shedding from epithelial cells in epididymis and prostate and endothelial cells of lung capillaries. The differences in local conformation of ACE could be the base for the generation of mAbs distingushing tissue-specific ACEs.

## Introduction

Angiotensin I-converting enzyme (ACE, CD143) is a Zn^2+^ peptidyldipeptidase which plays key roles in the regulation of blood pressure and in the development of vascular pathology and remodeling. ACE is constitutively expressed on the surface of endothelial cells, epithelial and neuroepithelial cells and cells of the immune system (macrophages, dendritic cells, reviewed in [1–3].

In addition to membrane-bound ACE, blood, seminal fluid and other biological fluids contain a variable amount of soluble ACE. Blood ACE likely originates from the vascular endothelium [4], mostly lung endothelial cells, because lung capillaries exhibit nearly 100% ACE expression compared to only 10–15% ACE-positive capillaries in the systemic circulation [5]. ACE enters the circulating pool via a proteolytic cleavage from the cell surface [6–7] by still unidentified membrane-bound ACE secretase [8].

Human seminal fluid ACE likely originates from glandular epithelial cells of epididymis and prostate, that express significant amount of somatic ACE [9–14]. Human seminal fluid contains 50-fold more ACE than blood [15–17]. However, the level of somatic ACE expression in male reproductive tract is comparable to somatic ACE expression in endothelial cells of capillaries [14, 18]. Therefore extremely high level of ACE in seminal fluid could be due to the higher ratio of the surface of epithelial cells producing ACE in the reproductive tract to the volume of seminal fluid than the ratio of the surface of ACE-producing endothelial cells of lung capillaries to the blood volume. Alternatively, it could be due to increased shedding of ACE from the surface of epithelial cells of epididymis and prostate in comparison to ACE shedding from endothelial cells.

There are at least two possible reasons of increased shedding: 1) Increased expression of ACE secretase in glandular epithelial cells of epididymis and prostate (but unfortunately the nature of ACE secretase is still unknown); 2) Different conformations of ACE on the surface of endothelial and epithelial cells, which, in turn, may lead to either different exposure of stalk region, where ACE secretase cleaves ACE from cell surface, or different regulation of ACE shedding from different cells by the presence of putative ACE-binding proteins/ACE effectors.

Species specificity of ACE is apparent; however, more subtle tissue specificity of the enzyme can influence ACE functions both *in vitro* and *in vivo*. We demonstrated recently that the pattern of mAbs binding to the conformational epitopes on the surface of ACE, “**conformational fingerprint of ACE**”, is an extremely sensitive marker of the local conformational changes in ACE molecule due to mutations, denaturation, inhibitor binding, presence of membrane anchor, etc. We proved the concept that the conformation of ACE is cell-and tissue-specific and stems likely from different post-translational modifications, including different glycosylation, on the example of ACE from endothelial cells and ACE from macrophages and dendritic cell of sarcoid granuloma. The ACE fingerprint, therefore, has a potential for the disclosure of the cells from which ACE originates [19].

We applied this approach here to detect structural (conformational) differences of ACEs originated from endothelial cells (lung ACE) and epithelial cells (male reproductive tract). We believe that structural differences in ACEs from different organs, which we found, could be a base for the development of antibodies, which will distinguish ACEs shed to the blood circulation from different organs by their origin.

## Experimental Section

### ACEs from different sources

The work was carried out in accordance with The Code of Ethics of World Medical Association (Declaration of Helsinki) and was approved by the Institutional Review Boards of the Bakulev Center of Cardiovascular Surgery, Moscow University and the University of Illinois at Chicago. Written informed consent from the donor or the next of kin was obtained for the use of this sample in research. Human citrated plasma, seminal fluid, lung, prostate and epididymis tissue homogenates were used as sources of somatic two-domain ACE. Lung and seminal fluid ACEs were purified using lisinopril affinity chromatography exactly as in [20]. Estimation of the percentage of ACE molecules carrying hydrophobic anchor (membrane form) in lung ACE preparation was performed by phase separation in Triton X-114 solution [21].

### ACE activity assay

ACE activity in blood plasma, seminal fluid, homogenates of human organs was measured using a fluorimetric assay with two ACE substrates, 2 mM Z-Phe-His-Leu (ZPHL) and 5 mM Hip-His-Leu (HHL), at pH 8.3 [22]. Briefly, 20 ul aliquots of samples were added to 200 ul of ACE substrate and incubated for the appropriate time at 37°C. The His-Leu product was quantified fluorimetrically via complexing with *o*-phtaldialdehyde. Inhibition of ACE activity by ACE inhibitors was performed by incubation of ACE (2 nM) with up to 0.1 uM enalaprilat and up to 1 uM teprotide, at pH 7.5 for one hour and then measurement of residual ACE activity with 0.2 mM ZPHL as a substrate at the same pH value. Inhibition of ACE activity with anti-catalytic anti-ACE mAbs was performed by 10 ug/ul mAbs as described in [23].

### Immunological characterization of ACEs

Ninety six-well plates (Corning, Corning, NY) were coated with anti-ACE mAbs via goat anti-mouse IgG (Pierce, Rockford, IL) bridge [24] and incubated with different sources of ACE, which were equilibrated for ACE activity with ZPHL as a substrate. After washing off unbound ACE, plate-bound ACE activity was measured by adding a substrate for ACE (ZPHL) directly into the wells [24]. Sixteen mAbs to human ACE were generated in our lab [19], while mAb BB9 [25] was kindly provided by Paul J Simmons (then Brown Foundation of Molecular Medicine, University of Texas Health Science Center, Houston, TX, USA).

Immunochemical characterization of ACE in the presence of different effectors was performed as described above, ACE solution being mixed with a solution of effectors just before application on the plate wells. The effectors used were heat-inactivated citrated plasma, heat-inactivated seminal fluid, 3 kDa filtrate of human citrated plasma, albumin, and bilirubin. Heat-inactivated plasma and seminal fluid were prepared by heating at 65°C for 45 min and checked for the absence of ACE activity; 3 kDa filtrate was prepared by filtration of human citrated plasma on Vivaspin 500 MWCO 3000 concentrator (GE Health Care, Sartorius Corp., Bohemia, NY) at 4°C and 12,000 g for 2 h.

### Immune responce to ACE from seminal fluid

Mice were immunized five times with pure seminal fluid ACE at 10ug per i.p. injection. The sera were tested against this immunogen in ELISA and against two ACEs, pure seminal fluid ACE and pure lung ACE, in plate precipitation assay. Upon reaching antibody titers 5x10^5^-10^6^, as measured by ELISA, mice were boosted and isolated splenocytes were fused to mouse myeloma cells 653-Ag8. The resulting populations grown in 96-well plates were subjected to primary screening with two pure ACEs, seminal fluid ACE and lung ACE, in plate precipitation assay in simultaneous experiments.

### Estimation of the size of the surface influenced by oligosaccharide

Sialylated biantennary complex oligosaccharide was modeled with the program Insight II (Accelrys Inc., San Diego, CA) and attached to the each potential site of glycosylation on the surface of the C domain (PDB 108A) of ACE. Molecular dynamics during 500 psec was applied for the estimation of the movement of the glycan and the neighboring amino acid residues within 15 Å from definite Asn residue.

### Desialylation of ACE

Desialylation of ACE was performed by neuraminidase from Vibrio Cholerae (Sigma-Aldrich, MO) in 0.1 M MES buffer, pH 6.0, containing 0.15 M KCl and 0.4 mM CaCl_2_, at 25°C. The process was controlled by ion-exchanged chromatography of 1 uM solutions of native and desialylated ACEs on Agilent1100 chromatograph with electrochemical cell. Chromatograms of native and desialylated ACEs were treated by the program Agilent Chemstation A.09.01 (Santa Clara, CA). For the calibration curve, solutions of neuraminic acids of 1, 10, and 20 uM were used.

### Protein digestions and mass spectrometry

The lung ACE and seminal fluid ACE were subjected to electrophoresis in denaturing conditions. For each ACE sample, two protein bands on an electrophoretic gel were obtained, which were further subjected to mass spectrometry peptide fingerprinting as follows. A piece of ACE band from electrophoretic gel was transferred into a test tube, washed from the dye, and then the protein was digested by trypsin as is described elsewhere [26]. Peptides were extracted with 30% acetonitrile containing 0.1% trifluoroacetic acid and subjected to MALDI-TOF mass spectrometry. All mass spectra were obtained on a time-of-flight MALDI-TOF UltrafleXtreme mass spectrometer (Bruker Daltonics, Germany) using 2,5-dihydroxybenzoic acid as a matrix. The data were analyzed by MASCOT program (www.matrixscience.com) with the use of SWISS-PROT database, and then using the ExPASy online tools, FindPept (www.expasy.org/findpept) and GlycoMod (web.expasy.org/glycomod). The FindPept tool was used to find additional matching to mass spectrometry data, peptides, such as C-terminal (unspecific) tryptic peptides which could not be discriminated by the MASCOT program. Non-matched peaks from the mass spectra were further analyzed with the GlycoMod tool in order to find possible glycopeptides and N-linked glycans in the ACE. The possibility of cysteine modification with acrylamide and methionine oxidation, which are typical variable modifications in mass spectrometry of proteins, was included in the analysis as well as the possibility for one or two missed cleavages with trypsin. Since a search with the GlycoMod tool is based on a comparison of experimental masses of glycopeptides with theoretical ones, its results sometimes contain false positive hits (displaying rather exotic glycan structures). So, finally only those N-linked glycan structures were taken into consideration and further manual analysis that matched the oligosaccharide compositions contained in the UniCarbKB database.

## Results and Discussion

### Immunological characterization of ACEs purified from human lung and seminal fluid

**Fig 1:**
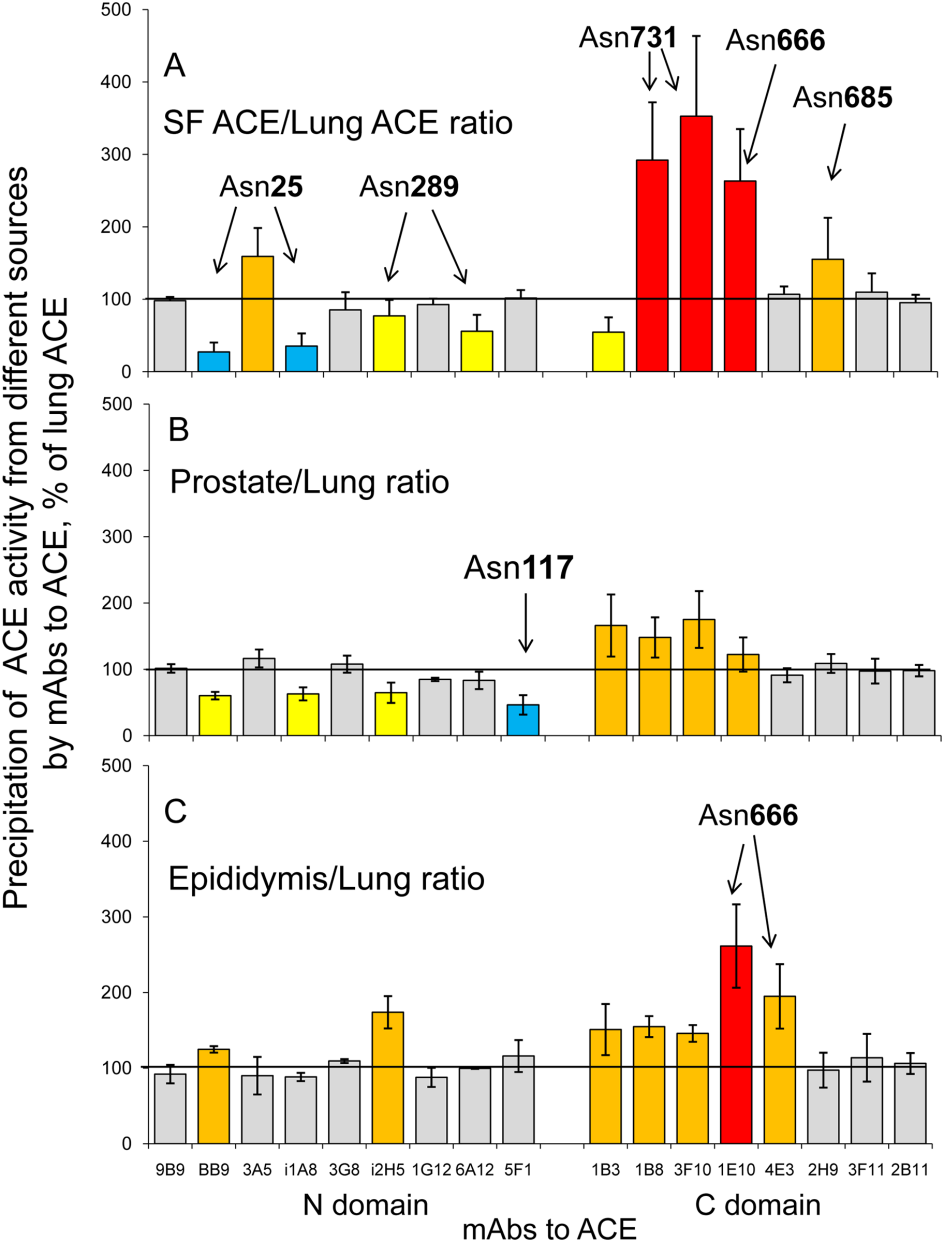
Immunological characterization of different ACEs. Conformational fingerprinting of different ACEs with a set of mAbs to ACE. Seventeen monoclonal antibodies (mAbs) were used to precipitate ACE from different sources. Immunoprecipitated ACE activity is presented as a normalized value (“binding ratio” expressed as a percentage of the activity of ACE from one source to that from another source) to highlight differences in immunoprecipitation pattern (“conformational fingerprint”) among different ACE variants. **(A)**. The ratio of precipitated activity of pure seminal fluid ACE to that of pure lung ACE. **B-C**. The ratio of precipitated activity of ACE from prostate homogenate (**B**) or epididymis homogenate (**C**) to that of lung homogenate. Data are presented as a mean of at least 3 independent experiments. Ratios increased more than by 20% are highlighted in orange and more than by 100% are highlighted in red. Ratios decreased more than by 20% are highlighted in yellow, more than by 50% in deep blue.

We characterized the conformation of pure lung and seminal fluid ACE, using a panel of mAbs directed against 17 different epitopes located on the N and C domains of catalytically active human ACE - “conformational fingerprint of ACE”[19]. As apparent in Fig 1A, the immunoprecipitation profile of pure ACEs from seminal fluid and lung dramatically differed. Because we mapped the epitopes for all these mAbs to human ACE [27–31] we can make a conclusion that seminal fluid ACE (originated from epithelial cells of epididymis and prostate) and lung ACE (originated from endothelial cells) exhibit different conformations of their surface. Most probable, these differences are caused by different glycosylation of ACE from different cells.

Tissue- and cell-specific post-translational modifications (PTM) are common for various proteins [32–36]. PTMs precisely regulate the functions of proteins by inducing conformational changes which subtly or dramatically alter the protein surface or its overall tertiary structure. The most common PTM is glycosylation, however, phosphorylation, methylation, acetylation, deamidation, etc. are also widespread [32, 35–37].

Somatic ACE represents a characteristic N-glycosylated glycoprotein, exact glycan structures of which, as well as exact locations of actually present oligosaccharide chains on the surface of the protein globule, can vary with different protein sources. The sequence of human somatic ACE contains 17 potential sites for N-glycosylation [38]. The structure and exact positions of glycan moiety in human somatic ACE from different tissues are still poorly investigated. It was reported that seven of the 17 potential sites are glycosylated in human seminal plasma ACE (Asn9, in particular), the majority of glycans belonging to biantennary structures [39]. Human kidney ACE was reported to have at least six N-glycosylated sites, residues 9, 25, 82, 117, 480, and 913 [26]. Human plasma proteomics revealed three glycosylation sites in blood ACE, Asn480, 666, and 685 [40].

It was shown previously that somatic ACE contains mostly complex-type oligosaccharides [26, 39]. Modeling of the structure of the common sialylated biantennary complex oligosaccharide and subsequent molecular dynamics of single-domain ACE with attached oligosaccharide allowed us to show that the contact of the oligosaccharide chain with the surface of the enzyme can occur within the area about 200–300 Å. As epitopes for mAbs are usually 400–750 Å [27], it is obvious that the presence or absence of the oligosaccharide (as well as its definite structure, i.e. the number of antennae, sialylation, fucosylation, etc.) within an epitope can hugely influence on mAbs binding.

Seminal fluid ACE and lung ACE possess rather equal masses as was shown by SDS-PAGE (not shown) which indicates on the absence of major differences in the degree of glycosylation of these ACEs. However, some evidence of different modes of glycosylation of lung ACE and seminal fluid ACE was obtained by the analysis of the action of neuraminidase on both enzymes. It was found that while desialylation of seminal fluid ACE did not result in the appearance of any neuraminic acid on the ion-exchange chromatogram, desialylation of lung ACE resulted in the appearance of 5 neuraminic acid residues per molecule of the enzyme (not shown). It is known that human lung ACE is characterized by a lower pI value (5.1) compared to that of kidney ACE (5.2) which was attributed to a bigger number of neuraminic acid residues in lung ACE glycans [41]. On the other hand, the number of neuraminic acid residues per molecule of kidney ACE was reported to be equal to 5.9 [42]. So, based on a lower pI value of lung enzyme, we might expect much larger content of neuraminic acid residues per molecule of lung ACE than 5 observed in our work. Moreover, we did not found any neuraminic acid residues for ACE from kidney as well (not shown). It seems, that desialylation in experimental conditions was not complete which resulted in underestimated values, if any, of neuraminic acid residues per ACE molecule. Thus, the difference in neuraminic acid residues content between seminal fluid ACE and lung ACE can not be considered as quantitative finding but rather an indication that the degree of sialylation of seminal fluid ACE is much less than that of lung ACE. The molecular basis for this phenomenon could be that, while the composition of the oligosaccharide chains in lung ACE is still unknown, seminal fluid ACE contains five complex-type glycans, which can contain sialic acid residues, while two other glycans in this ACE represent oligomannose-type glycans [39]. In addition, it is interesting that from 5 sialic acid residues found for lung ACE, three residues corresponded to N-acetyl-neuraminic acid whereas two residues represented N-glycolyl-neuraminic acid, which was reported earlier to be abundant in testicular ACE but not found in kidney ACE [39].

An analysis of the conformational fingerprint of lung ACE and seminal fluid ACE (Fig 1A) allowed us to suggest that different glycosylation of ACEs from different cells might occur in the following glycosylation sites on ACE protein globule: Asn25 in the epitopes for mAbs BB9, 3A5 and i1A8, Asn289 in the epitopes for mAbs i2H5 and 6A12 on the N domain, Asn666 in the epitopes for 1E10 and 4E3, and Asn685 in the epitope for 2H9 on the C domain.

**Fig 2:**
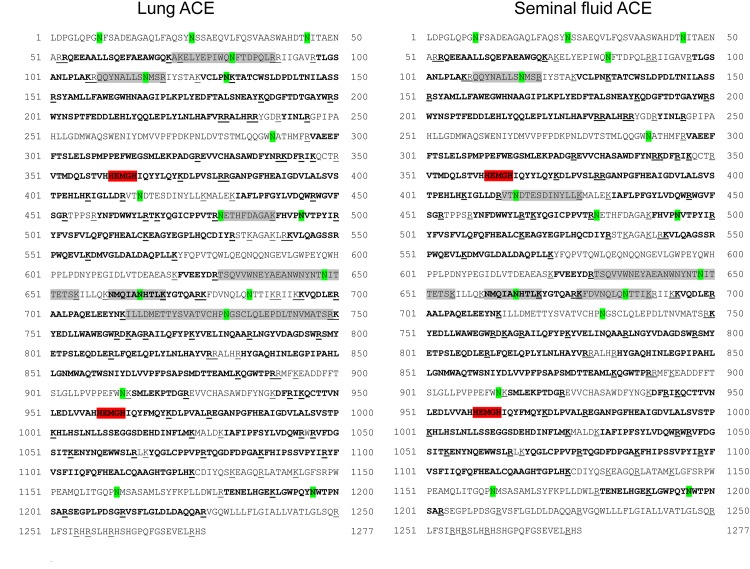
Amino acid sequences of the lung and seminal fluid ACEs. Peptides identified by MALDI TOF MS are shown in bold; potential sites of trypsin cleavage are underlined; potential glycosylation sites are marked by green; zinc-recognizing motives are marked by red; putative glycopeptides are shaded.

As for the dramatic difference in binding of mAbs 1B8 and 3F10 to seminal fluid ACE and lung ACE, it might be explained by the difference in glycosylation of Asn731, which is in the center of the epitopes for these two mAbs on the C domain of ACE [30] or, alternatively, by the presence of transmembrane anchor in some molecules of lung ACE, which influences the binding of these very mAbs to ACE [19, 30]. The presence of such molecules was demonstrated by MALDI TOF analysis which revealed in lung ACE digested with trypsin a presence of not only the C-terminal peptide 1190–1203 but also peptides 1204–1213 and even 1214–1227 (Table 1 and Fig 2). To estimate possible contamination of lung ACE with molecules carrying hydrophobic transmembrane anchor, we analyzed lung ACE preparation by phase separation in Triton X-114. About 90% of the enzyme was determined in water phase, while only 10% appeared in organic phase. Therefore, the proportion of ACE molecules with transmembrane-anchor in the pure lung ACE preparation is not high and we can conclude that the presence of anchor-containing molecules in lung enzyme unlikely contributes to the observed difference in the binding of mAbs to the C-region of ACE molecule.

**Table 1:** Observed [M+H]^+^ ions of unglycosylated peptides in the mass spectra of human ACE tryptic digests.

Lung ACE			Seminal fluid ACE		
Peptide	Observed *m/z*	Calculated *m/z*	Peptide	Observed *m/z*	Calculated *m/z*
53–71^c^	2191.1	2191.1	53–71^c^	2191.1	2191.1
54–71	2035.0	2035.0	54–71	2035.0	2035.0
97–107	1084.6	1084.6	97–107	1084.7	1084.6
**127–132** ^a^	744.4	744.4	**127–132** ^a^	744.4	744.4
133–151^a^	2135.1	2135.0	133–151^a^	2135.1	2135.0
152–187	4117.1	4117.0	152–187	4117.2	4117.0
152–187^b^	4133.0	4133.0	152–187^b^	4132.9	4133.0
188–199	1416.6	1416.6	188–199	1416.6	1416.6
200–230	3837.9	3837.9	200–230	3837.8	3837.9
231–236^c^	808.4	808.5	231–236^c^	808.5	808.5
241–245	678.4	678.4	241–245	678.4	678.4
296–326	3526.7	3526.7	296–326	3526.7	3526.7
296–326^b^	3542.7	3542.6	296–326^b^	3542.7	3542.6
327–340^a^	1767.8	1767.8	327–340^a^	1767.8	1767.8
327–341^a^ ^,^ ^c^	1895.9	1895.9	327–341^a^ ^,^ ^c^	1895.9	1895.9
342–346^c^	678.4	678.4	342–346^c^	678.4	678.4
351–373	2821.4	2821.3	351–373	2821.3	2821.3
351–373^b^	2837.4	2837.3	351–373^b^	2837.3	2837.3
374–380	799.5	799.5	374–380	799.5	799.5
381–407^c^	2852.5	2852.5	381–407^c^	2852.5	2852.5
382–407	2696.4	2696.4	382–407	2696.4	2696.4
408–413	686.4	686.4	408–413	686.5	686.4
433–446	1724.9	1724.9	433–446	1724.9	1724.9
447–453	808.4	808.4	447–453	808.4	808.4
459–467	1362.6	1362.6	459–467	1362.7	1362.6
468–479^c^	1362.7	1362.7	468–479^c^	1362.7	1362.7
470–479	1133.6	1133.6	470–479	1133.6	1133.6
**490–500**	1342.7	1342.7	**490–500**	1342.8	1342.7
			**490–517** ^c^	3429.5	3429.8
501–532^c^	3837.9	3837.8	501–532^c^	3837.8	3837.8
518–532^a^	1821.8	1821.8	518–532^a^	1821.8	1821.8
542–557^c^	1826.0	1826.0	542–557^c^	1826.0	1826.0
543–557	1697.9	1697.9	543–557	1697.9	1697.9
558–572	1598.9	1598.8	558–572	1598.9	1598.8
623–629	957.4	957.4	623–629	957.5	957.4
**661–677** ^c^	1973.9	1974.0	**661–677** ^c^	1973.8	1974.0
671–676	695.4	695.3	671–676	695.4	695.3
694–700^c^	887.5	887.5	694–700^c^	887.5	887.5
701–713	1475.8	1475.7	701–713	1475.8	1475.7
750–762^c^	1751.9	1751.9	750–762^c^	1751.9	1751.9
751–762	1623.8	1623.8	751–762	1623.8	1623.8
751–767^c^	2151.1	2151.0	751–767^c^	2151.1	2151.0
768–775	979.6	979.6	768–775	979.6	979.6
776–785	1176.7	1176.6	776–785	1176.7	1176.6
786–797	1352.6	1352.6	786–797	1352.6	1352.6
798–811	1697.9	1697.8	798–811	1697.9	1697.8
798–811^b^	1713.9	1713.8	798–811^b^	1713.8	1713.8
812–828	2117.2	2117.2	812–828	2117.2	2117.2
834–883	5562.0	5561.7	834–883	5561.3	5561.7
884–889	744.4	744.4	884–889	744.4	744.4
915–924	1133.6	1133.6	915–924	1133.6	1133.6
940–944^c^	678.4	678.4	940–944^c^	678.4	678.4
945–971^a^	3305.6	3305.6	945–971^a^	3305.5	3305.6
972–978	783.5	783.5	972–978	783.5	783.5
979–1001	2309.3	2309.2	979–1001	2309.2	2309.2
1002–1025	2693.3	2693.3	1002–1025	2693.3	2693.3
1031–1044	1754.9	1754.9	1031–1044	1755.0	1754.9
1045–1077^c^	4066.1	4066.1	1045–1077^c^	4066.0	4066.1
1047–1054	866.5	866.5	1047–1054	866.5	866.5
1055–1065	1524.7	1524.7	1055–1065	1524.7	1524.7
1068–1077	1129.6	1129.6	1068–1077	1129.6	1129.6
1078–1087	1035.5	1035.5	1078–1087	1035.5	1035.5
1088–1098	1315.7	1315.7	1088–1098	1315.7	1315.7
1099–1125	3089.5	3089.5	1099–1125	3089.4	3089.5
1181–1189	1056.5	1056.5	1181–1189	1056.5	1056.5
			**1190–1202**	1533.7	1533.8
**1190–1203**	1689.8	1689.8	**1190–1203**	1689.8	1689.8
1204–1213	1014.5	1014.5			
1214–1227	1532.8	1532.8			

^a^ Acrylamide adduct on cysteine.

^b^ Oxidized methionine.

^c^ Contains one or two missed cleavage(s) by trypsin.

Peptides that contain potential N-glycosylation sites are shown in bold.

In order to analyze the reasons for the differences in mAbs 1B8 and 3F10 binding with lung and seminal fluid ACE further we compared conformational fingerprint of ACE from lung homogenate with that from epididymis and prostate homogenates (Fig 1B and 1C), which should have similar proportion of anchor-containing ACE molecules. This comparison clearly demonstrates remarkable differences in conformation of ACE from lung and ACE from both prostate and epididymis. Better binding of mAbs 1B8 and 3F10 to prostate and epididymal ACE, compared to lung ACE, reproduces better binding of these mAbs to pure seminal fluid ACE compared to pure lung ACE and most probably indicates different glycosylation (extent or type) of Asn731 in ACE from the epithelial cells of prostate and epididymis than in lung ACE from endothelial cells (Fig 1B and 1C).

The enhanced binding of these two mAbs, as well as mAb 1B3 (directed to the epitope on the stalk region [43–44]), to epididymal and prostate ACE allows us to suggest that better access of the stalk region to these mAbs may indicate on the better access of the stalk region in ACE on the surface of epithelial cells of prostate and epididymis for ACE secretase as well and may result in higher rate of shedding

Moreover, a comparison of the conformational fingerprints of epididymal and prostate ACE with their seminal fluid ACE counterpart indicates that seminal fluid ACE could represent a mixture of ACE originated from epididymis and ACE originated from prostate. In support of this suggestion, we discovered by MALDI-TOF analysis of seminal fluid ACE digested with trypsin (Table 1) a presence of two C-terminal peptides, a peptide with molecular mass 1533.8 (found among matching peptides for unspecific cleavage by FindPept tool) which corresponds to the peptide 1190–1202 (calculated mass 1533.7) reported previously [45] and a peptide with molecular mass 1689.8 which corresponds to the peptide 1190–1203 (calculated mass 1689.8). So, it is possible that the seminal fluid contains two somatic ACE forms—shorter form of ACE (1202 residues) and common form (1203 residues), which can be produced differently by epididymis and prostate or, alternatively, the shorter form could be the result of the secondary carboxypeptidase processing as suggested in [45].

Measurement of ACE activity in tissues homogenates prepared in equal conditions (tissue:buffer = 1:9) revealed that prostate exhibited much lower content of ACE (44 mU/g), than epididymis (720 mU/g), which ACE activity was comparable to that in lung—1063 mU/g with ZPHL as a substrate. These values are in accordance with lower ACE protein content in prostate than in epididymis reported earlier [11–12] and confirmed recently in the Human Protein Atlas (www.proteinatlas.org), which demonstrates that epididymis exhibits at least 6-fold more ACE expression than prostate. However, higher content of ACE in epididymis is likely compensated by higher mass of prostate which leads to pretty equal content of ACE in both organs [11] and perhaps explains why ACE in seminal fluid is considered to originate equally from both organs [16]. This conclusion is supported by the comparison of conformational fingerprints of prostate, epididymis and seminal fluid ACEs (Fig 1). It seems that the pattern of mAbs binding to pure seminal fluid ACE represents a mixture of patterns of mAbs binding to prostate and epididymal ACE. Thus, epididymal ACE, but not prostate ACE, exhibited enhanced binding of mAb 1E10 to the C domain of ACE, which is also characteristic for seminal fluid ACE. As opposite, prostate ACE is characterized by lowered binding of mAbs BB9, i1A8, and i2H5 to the N domain of ACE, which is characteristic for seminal fluid ACE as well (Fig 1).

ACE in prostate homogenate, however, is also characterized by a very low binding of mAb 5F1, while the binding of this mAb to ACE in epididymal homogenate and to pure seminal fluid ACE are rather high. The epitope for this mAb contains potential site of glycosylation, Asn117 [29]. Moreover, another site of glycosylation, Asn480, is located close to the epitope for mAb5F1 [29]. It was shown earlier [40] that, at least, in serum ACE Asn480 is glycosylated. So, we could suggest that glycosylation of Asn117 and/or Asn480 is dramatically different in prostate ACE in comparison to epididymal and lung ACEs (Fig 1B and 1C).

As a whole, the results allow us to assume that seminal fluid ACE originates likely equally from epididymis and prostate glandular epithelial cells.

### Identification of N-linked glycosylation sites in lung and seminal fluid ACEs

Somatic ACEs isolated from human lung and seminal fluid were digested with trypsin and the resulting peptides were analyzed using MALDI TOF MS. After a primary search of matching tryptic peptides with the MASCOT program, the protein sequence coverage was about 50% in both cases. After finding additional matching peptides using the FindPept tool, the sequence coverage became higher, 61% for the lung ACE and 59% for seminal fluid enzyme. Unglycosylated peptides identified by the FindPept tool in the tryptic digest of the lung ACE are listed in Table 1 and shown in Fig 2; four of these peptides contain potential glycosylation sites, Asn131, 494, 666, and 1196. The same four peptides were found in the trypsin digest of seminal fluid ACE (Table 1). It should be noted, however, that the intensity of the MS peak corresponding to the peptide 661–677, containing Asn666 as a potential glycosylation site, was extremely small, especially in the case of the lung ACE. It casts doubt on the classification of this peptide to the unglycosylated ones.

Analysis of the mass spectra data with the GlycoMod tool allowed revealing several peaks matching by mass potential glycopeptides in the lung ACE and seminal fluid ACE. After expert revision, we limited the number of putative glycopeptides by several ones with the most favorable structures reported in UniCarbKB. The results are presented in Table 2. It is seen that in some cases the interpretation could be rather equivocal, as the data could be attributed to different peptides within amino acid sequence of ACE. In a few cases, an observed peak could be related to the glycopeptides with the same peptide structure but with different glycan moieties.

**Table 2:** N-glycosylated peptides and glycan structures found by the GlycoMod tool in ACE from lung and seminal fluid using MS data.

**Lung ACE**				
N-Glycosylation site	Peptide	Observed *m/z*	Calculated *m/z*	Putative glycan structures
Asn82	Ala72-Arg89	3853.9	3853.7	(HexNAc)_2_(NeuAc)_1_+(Man)_3_(GlcNAc)_2_
Asn117	Gln109-Arg120	3030.4	3030.3	(HexNAc)_2_(NeuAc)_1_+(Man)_3_(GlcNAc)_2_
Asn480	Asn480-Lys489		2468.0	(Hex)_3_+(Man)_3_(GlcNAc)_2_
and/or		2468.1		
Asn666	Asn661-Lys670		2468.1	(HexNAc)_2_+(Man)_3_(GlcNAc)_2_
				(Hex)_5_(HexNAc)_4_(NeuAc)_1_+(Man)_3_(GlcNAc)_2_ and/or
Asn648	Thr630-Lys655		5885.4	(Hex)_4_(HexNAc)_4_(Deoxyhexose)_1_(NeuAc)_1_+(Man)_3_(GlcNAc)_2_
and/or		5885.4		
Asn731	Ile714-Arg749		5885.6	(Hex)_4_(HexNAc)_2_+(Man)_3_(GlcNAc)_2_ and/or
				(Hex)_3_(HexNAc)_2_(Deoxyhexose)_1_+(Man)_3_(GlcNAc)_2_
**Seminal fluid ACE**				
N-Glycosylation site	Peptide	Observed *m/z*	Calculated *m/z*	Putative glycan structures
Asn117	Gln109-Arg120	5469.1	5469.1	(Hex)_5_(HexNAc)_5_(Deoxyhexose)_1_(NeuAc)_4_+(Man)_3_(GlcNAc)_2_
Asn416	Val414-Lys427	3288.5	3288.5	(Hex)_1_(HexNAc)_3_+(Man)_3_(GlcNAc)_2_
Asn648	Thr630-Lys655	5885.2	5885.4	(Hex)_5_(HexNAc)_4_(NeuAc)_1_+(Man)_3_(GlcNAc)_2_ and/or
				(Hex)_4_(HexNAc)_4_(Deoxyhexose)_1_(NeuAc)_1_+(Man)_3_(GlcNAc)_2_
Asn666	Asn661-Lys670	3245.4	3245.3	(Hex)_3_(HexNAc)_2_(NeuAc)_1_+(Man)_3_(GlcNAc)_2_ and/or
				(Hex)_2_(HexNAc)_2_(Deoxyhexose)_1_(NeuAc)_1_+(Man)_3_(GlcNAc)_2_
Asn685	Lys677-Lys689	2765.3	2765.3	(Hex)_2_+(Man)_3_(GlcNAc)_2_

Nevertheless, we could state that asparagins 82, 117, 480, 648, 666, and 731 could be glycosylated in human lung ACE. The glycosylated Asn82, 117, and 480 have been found previously in human kidney ACE [26], while the glycosylated Asn480, 666, and 685 have been reported to be present in human plasma ACE [40]. As the source of plasma ACE is mainly lung ACE [4–5], we could assume that Asn685 in lung ACE may also be glycosylated. It is worth noting that the peptide 661–677 containing Asn666 was also found among unglycosylated peptides in the tryptic digest of the lung ACE (Table 1), although the intensity of the corresponding MS peak was very small. Thus, we could not consider this definite N-glycosylation site Asn666 as fully glycosylated, but rather as partially glycosylated. The peptides containing Asn82, 117, 480, 648, and 731 were not found among unglycosylated peptides. So, these sites could be fully glycosylated. There is still some uncertainty in referring the peptides with observed *m/z* 2468.1 to the peptides containing Asn480 or Asn666, as well as peptide with *m/z* 5885.4 to the peptides containing either Asn648 or Asn731. The putative N-glycosylation site Asn648 is not attributed to any known epitope for mAbs to ACE, while the N-glycosylation site Asn731 is a part of the epitopes for mAbs 1B8 and 3F10 to the C domain of ACE (Fig 2). The remarkable difference in the efficiency of these mAbs binding to the lung ACE and seminal ACE (Fig 1) convincingly shows that the glycosylation of this definite Asn731 is different in the lung ACE and seminal fluid ACE.

Similarly, asparagins in positions Asn117, 416, 648, 666, and 685 could be glycosylated in human seminal fluid ACE. The peptide 661–677 containing Asn666 was also found among unglycosylated peptides (Table 1), although the intensity of the corresponding MS peak was very small (as in the case of the lung ACE), therefore, this site could be partially glycosylated. Other Asn residues can be considered as fully glycosylated.

Comparison of the data obtained for lung and seminal fluid ACEs shows that both enzymes contain fully glycosylated Asn117, however, the structure of putative glycans is different, glycan from seminal fluid ACE exhibiting more branches. Both ACEs could contain fully glycosylated Asn648, the suggested glycans being similar in both cases. And, again, both ACEs could contain partially glycosylated Asn666, but the structures of suggested glycans in these ACE forms remarkably differ, glycans in seminal fluid ACE being more complicated. In addition, analysis of the tryptic digest of lung ACE revealed also Asn82, 480, and 731 as occupied N-glycosylation sites, while for seminal fluid ACE occupied N-linked glycosylation sites were found to be 416, and 685. The difference in N-glycosylation of ACE from different sources can be the reason for the remarkable difference in the efficacy of binding of some mAbs to these ACEs (Fig 1), i.e. differences in local ACE conformation.

**Fig 3:**
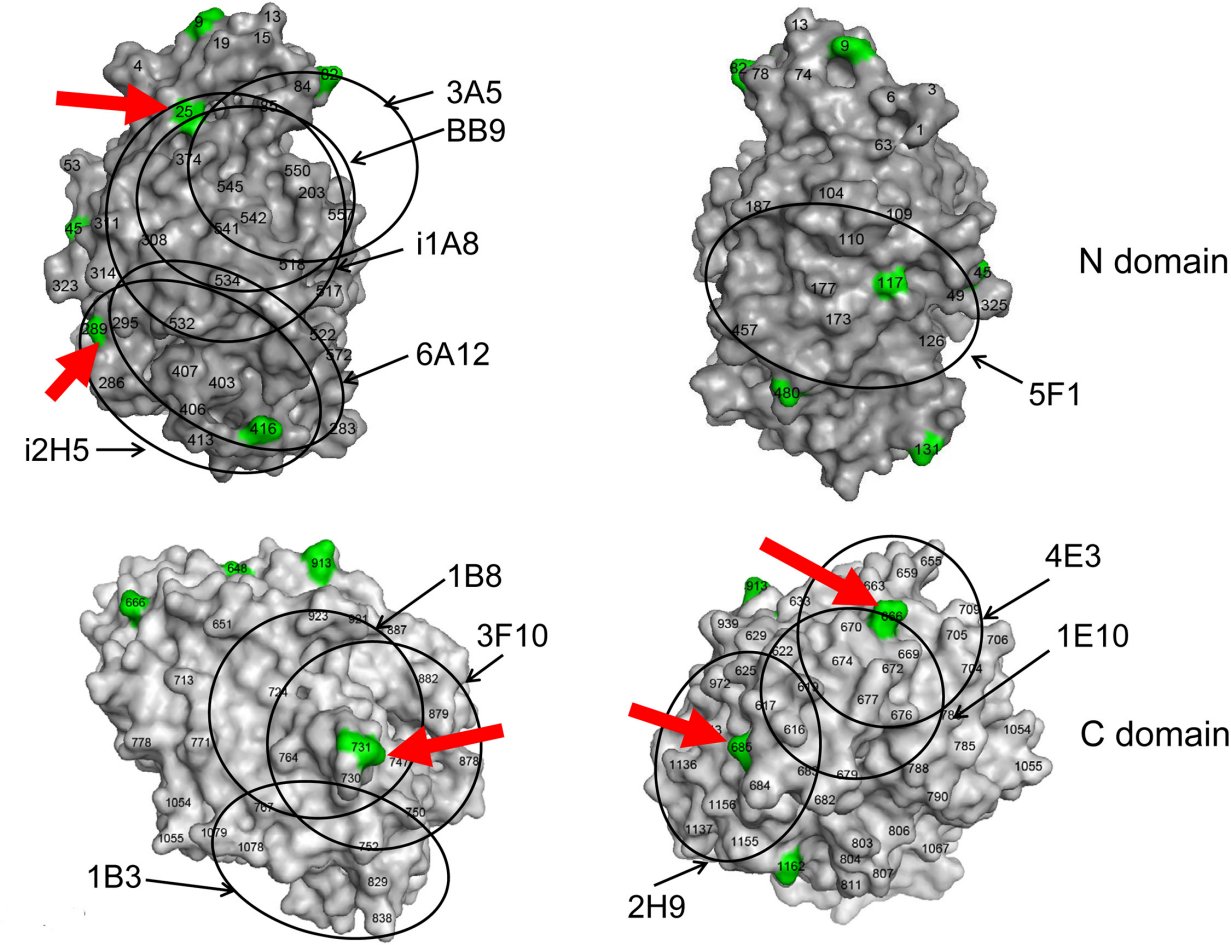
The structures of N and C domains of ACE with potential glycosylation sites and epitopes for mAbs. Human N domain structure was based on PDB P2C6N and C domain structure—based on PDB 1O86. The epitopes were marked on the N and C domains according to [27–31, 41]. The positions of the epitopes for some mAbs (12 out of 17) are shown by circles on both sides of domain globule. The potential sites of N-glycosylation, 9 on the N domain and 6 on the C domain, are marked by green; Asn494 on the N domain is not seen while Asn1196 is not present in structure of the C domain. The glycosylation sites which might be differently glycosylated in seminal fluid ACE and lung ACE are shown by arrows. Some amino acid residues are shown by numbers according to [38] for orientation.

The N-glycosylation sites on the N and C domains of human ACE are shown in Fig 3. In addition to the putative difference in ACE N-glycosylation revealed by the mass spectra analysis, different N-glycosylation in ACE originated from lung endothelial cells and in ACE from epithelial cells of prostate and epididymis can include Asn25, and 289 on the N domain, as indicated by the mAbs binding.

### Inhibition of seminal fluid and lung ACEs

**Fig 4:**
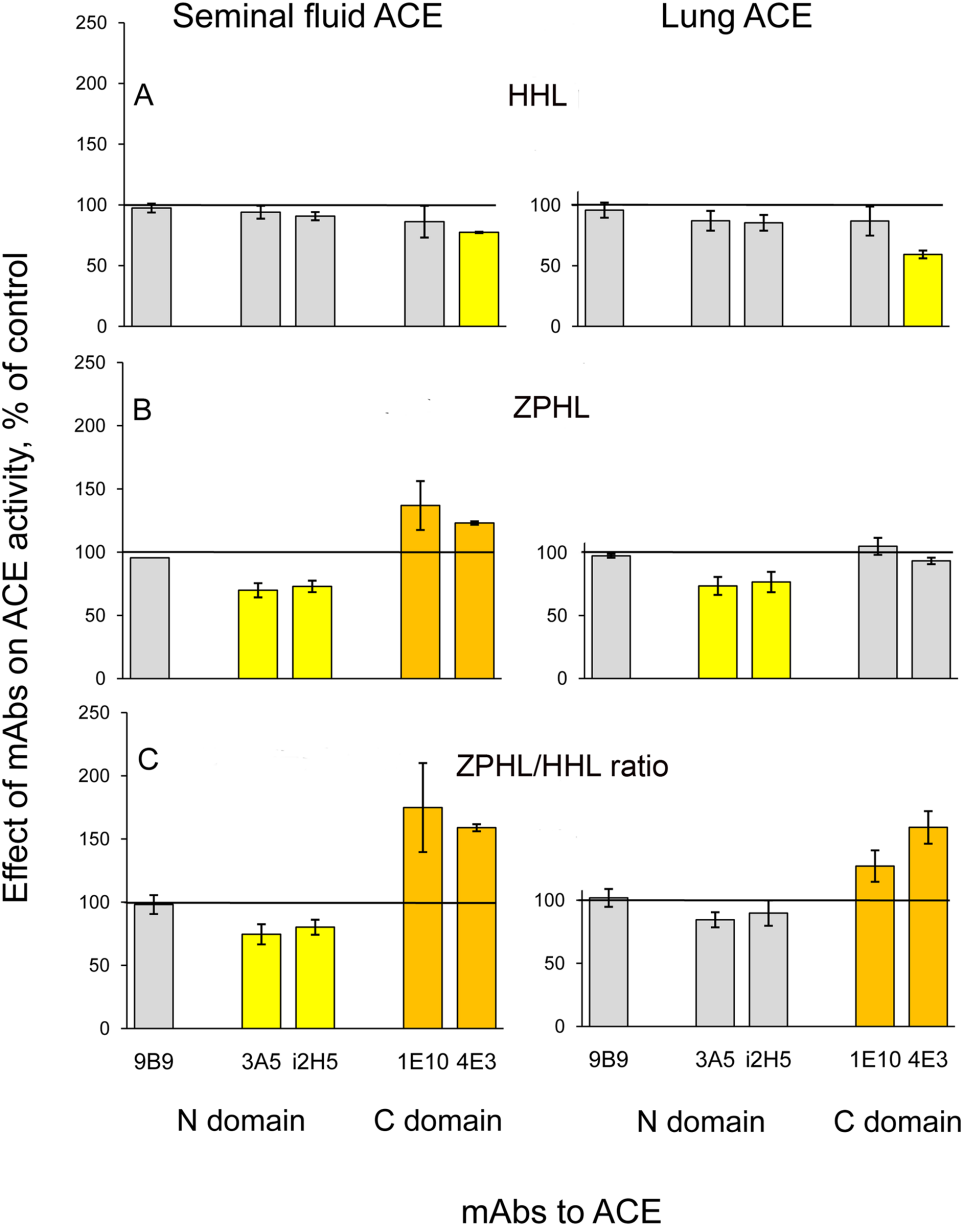
Effect of anti-catalytic mAbs on the activity of pure seminal fluid and lung ACEs. Pure seminal fluid and lung ACEs (5 mU/ml with ZPHL as a substrate) were incubated with mAbs (10 ug/ml), which are anti-catalytic for the N-domain active center, i2H5 and 3A5 [27], and for the C domain active center, 1E10 and 4E3 [30], of ACE. Residual ACE activity was determined with substrates HHL (**A**) and ZPHL (**B**) and is presented as the ratio of ACE activity in the presence of mAbs to that without mAbs. Data are also presented as ZPHL/HHL ratio of ACE activity in the presence of mAbs to that without mAbs (**C**). Results are the mean ± SD of 2–4 experiments, made in duplicates.

We examined the effect of ACE inhibitors, enalaprilat (analogue of tripeptide) and teprotide (nonapeptide), on enzymatic activity of pure lung and seminal fluid ACEs. Both ACEs were equally inhibited by ACE inhibitors with IC_50_ about 6 nM for enalaprilat and 10 nM for teprotide. We also tested the inhibitory effects of mAbs 3A5 and i2H5, anti-catalytic mAb to the N domain [24, 27], and mAbs 1E10 and 4E3, anti-catalytic mAbs to the C-domain [30] on ACE activity (Fig 4). Inhibitory effect of both mAbs to the N domain, 3A5 and i2H5, at tested concentrations was significant towards both ACEs only with a substrate ZPHL (Fig 4A and 4B). However, these mAbs can be considered as more effective towards seminal fluid ACE, as reflected by a more prominent decrease in the sensitive parameter ZPHL/HHL ratio (Fig 4C), this decrease indicating preferable inhibition of the N domain of ACE [22]. Inhibitory effect of mAbs, anti-catalytic to the C domain, was epitope-, substrate-, and tissue-specific. Both mAbs, 1E10 and 4E3, more effectively inhibited ACEs with HHL as a substrate, mAb 4E3 causing more noticeable effect than mAb 1E10 and more effectively inhibiting lung ACE activity (Fig 4A and 4B). However, an effect of these mAbs on ACE activity with ZPHL as a substrate was rather paradoxical: the binding of anti-catalytic mAbs to the C domain of seminal fluid ACE, but not to the lung ACE, caused reliable enhancement of ACE activity (Fig 4B). We can consider this fact as an indication of the putative conformational changes in the N domain of seminal fluid ACE, which are induced by the binding of mAbs to the adjacent C domain and accompanied by the increase of ACE N domain activity.

### Effect of potential ACE-binding partners on seminal and lung ACEs

How these remarkable differences in local conformation of seminal fluid and lung ACEs may be converted into different regulation of the functions of these ACEs, including the regulation of their shedding? We demonstrated recently that the rate of recombinant human ACE shedding from the surface of CHO-ACE, HEK-ACE and endothelial cells in the presence of bovine and human serum was dramatically diminished, up to 2-times. However, the extent of the serum effect varied depending on the type of serum and cells (unpublished observation).

**Fig 5:**
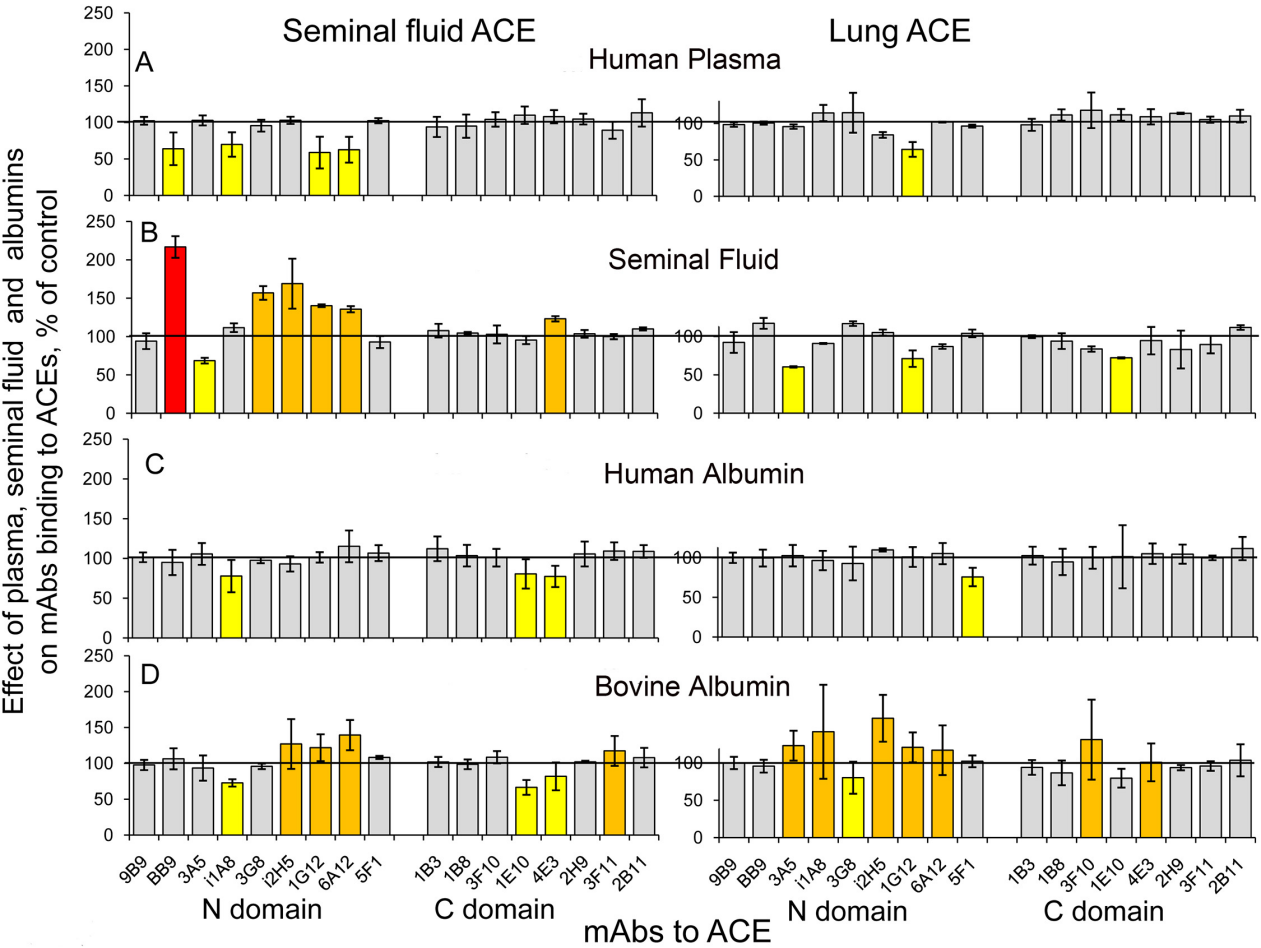
Effect of human plasma, seminal fluid and albumins on mAbs binding to ACEs. ACE activity immunoprecipitated by 17 mAbs to ACE (as in Fig 1) was presented as a normalized value (“binding ratio”) to highlight differences in immunoprecipitation pattern (“conformational fingerprint”) after adding heat-inactivated human citrated plasma, heat-inactivated seminal fluid, as well as human and bovine albumins to purified seminal fluid and lung ACEs with that without additives. **A-B**. Effect of 20% of heat-inactivated human plasma (**A**) and heat-inactivated seminal fluid (**B**); **C-D**. Effect of human (**C**) and bovine (**D**) albumins at concentrations of 8 mg/ml (similar to albumin concentration in 20% serum). Data are presented as in Fig 1.

Recently, it was found that albumin may bind to ACE [46–47]. Bearing in mind that albumin concentration in the seminal fluid (1 mg/ml) is 50-fold less than in the blood [48] it was very tempting to suggest that 50-fold increase in ACE concentration in seminal fluid is solely explained by 50-fold decrease in albumin concentration. Therefore, we estimated the effect of inactivated human plasma, inactivated seminal fluid, as well as pure human and bovine albumins, on mAbs binding to pure lung and seminal fluid ACEs (Fig 5).

Effect of inactivated human plasma on mAbs binding to two types of ACE differed significantly (Fig 5A), with seminal fluid ACE being more sensitive to the presence of plasma. Effect of inactivated seminal fluid on mAbs binding to two types of ACE also showed more prominent effect for seminal fluid ACE (Fig 5B), strengthening our suggestion that different conformations of these ACEs can participate in different regulation of the functions of ACEs on endothelial and epithelial cells. It is worth noting that only a small percentage of the overall effect of plasma on mAbs binding to ACE could be attributed to albumin (Fig 5C), namely, a decrease in binding of mAb i1A8, which is seen only for seminal fluid ACE. We also should state that an effect of pure albumins is strongly species specific (Fig 5C and 5D). While the effects of human and bovine albumins were rather equal on mAbs i1A8, 1E10, and 4E3 binding to seminal fluid ACE, only bovine albumin caused the increase of mAbs i2H5, 1G12, and 6A12 (with overlapping epitopes) binding to this enzyme. Alternatively, only human albumin caused the decrease of mAb 5F1 binding to lung ACE (without effect on the binding of other mAbs to lung ACE), whereas bovine ACE caused multiple conformational changes in this ACE.

### LMW component from blood also binds to ACE

**Fig 6:**
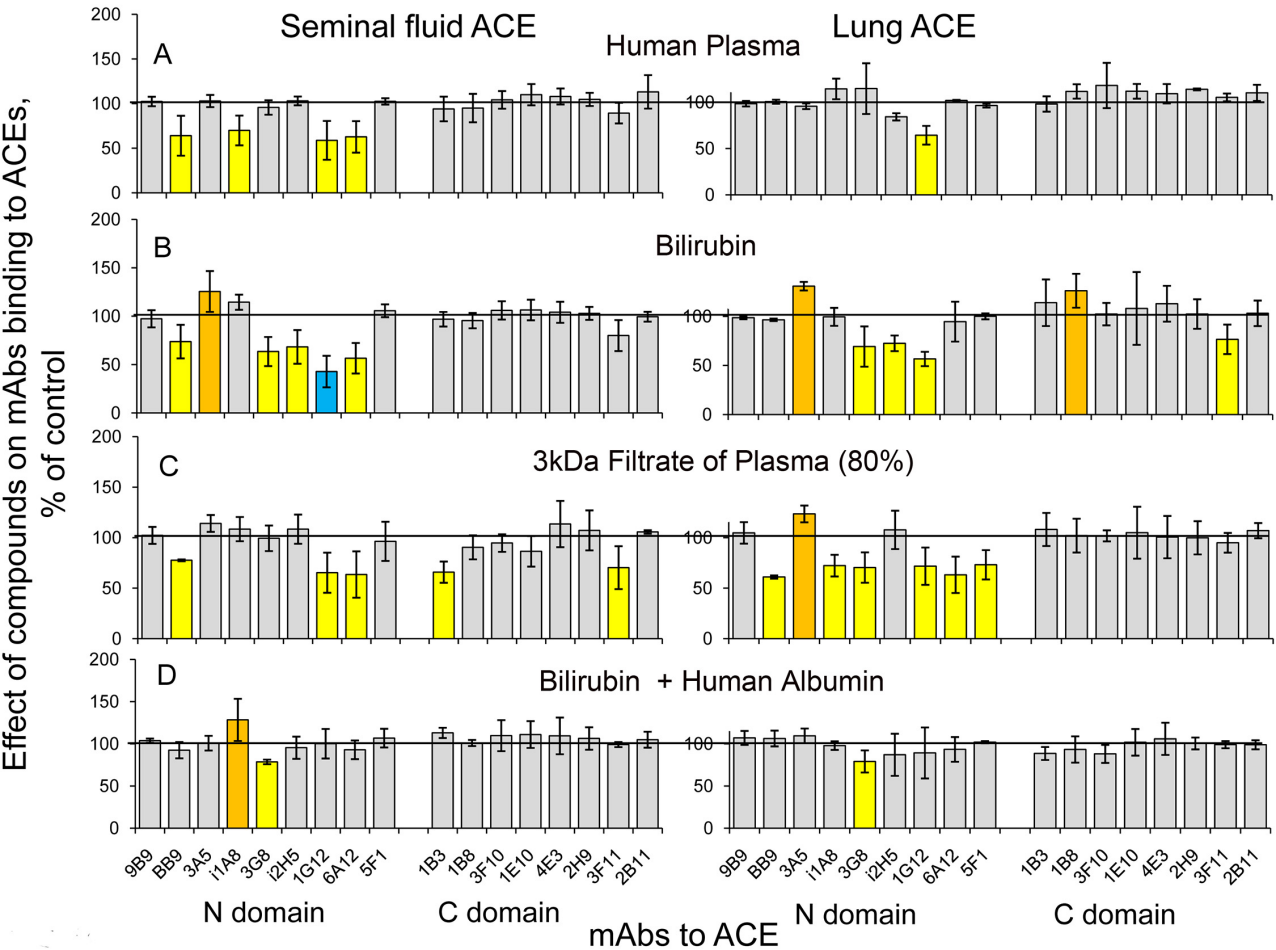
Effect of different additives on mAbs binding to seminal fluid and lung ACEs. ACE activity immunoprecipitated by 17 mAbs to ACE (as in Fig 1) was presented as a normalized value (“binding ratio”) to highlight differences in immunoprecipitation pattern (“conformational fingerprint”) after adding of tested compounds to purified seminal fluid and lung ACEs with that without additives. **(A)** Effect of 20% of human heat-inactivated plasma. **(B**) Effect of 80% 3 kDa filtrate of human citrated plasma. **C**-**D**. Effect of bilirubin (150 ug/ml) in the absence (**C**) or presence (**D**) of human albumin at 8 mg/ml concentration (which correspond to its concentration in 20% serum). Data are presented as in Fig 1.

We found that Low Molecular Weight (LMW) component from human plasma binds to human ACE (unpublished observation). Therefore, we tested an effect of human plasma 3kDa filtrate, containing LMW component (-s), on mAbs binding to both structurally different ACEs and showed that this filtrate had a strong (and different) effect on both types of ACE (Fig 6).

Comparison of the effect of 3 kDa human plasma filtrate (Fig 6B) with the effect of heat-inactivated human plasma (Fig 6A) clearly demonstrates that LMW components of human plasma exhibited more significant effect on mAbs binding to ACEs than proteins within this plasma.

We identified recently a bilirubin as one of the components of 3 kDa plasma filtrate which is able to bind to ACE in the region of the overlapping epitopes 1G12/6A12 (unpublished observation). Therefore, we tested an effect of this novel ACE-binding partner, bilirubin, on two different, tissue specific ACE types (alone or in combination with human albumin), seen on Fig 6C and 6D. Again, the effect of bilirubin on ACE was strongly tissue specific: significant effect of bilirubin on mAbs binding to the N domain of lung ACE, while less pronounced for seminal fluid ACE, and visible effect on the mAbs binding to the C domain of seminal fluid ACE without any effect on the C domain of the lung ACE (Fig 6C).

The fact that human albumin practically abolished an effect of bilirubin on mAbs binding to both ACEs (Fig 6D) shows that only unbound bilirubin (which represents only minor part (about 10%) of total bilirubin content in the blood [49] can bind to ACE.

Thus, significant differences in local conformation of lung ACE (originated from endothelial cells) and seminal fluid ACE (originated from epithelial cells of prostate and epididymis) allow us to suggest that possible regulation of ACE shedding from epithelial cells of male reproductive tract and from endothelial cells of capillaries (blood vessels) by the constituents of blood and seminal fluid may be different due to different effect of possible regulators of ACE shedding on the structurally different ACEs in these cells.

In addition, membrane-bound glycoproteins (including ACE) may often be shed into blood circulation as a by-product of the disease process. Recently an increase in blood ACE level was demonstrated for patients with epithelial ovarian carcinoma [50]. Likely, such an increase may be explained by an increase of local vascular permeability induced by the increased VEGF production by tumor tissues.

**Fig 7:**
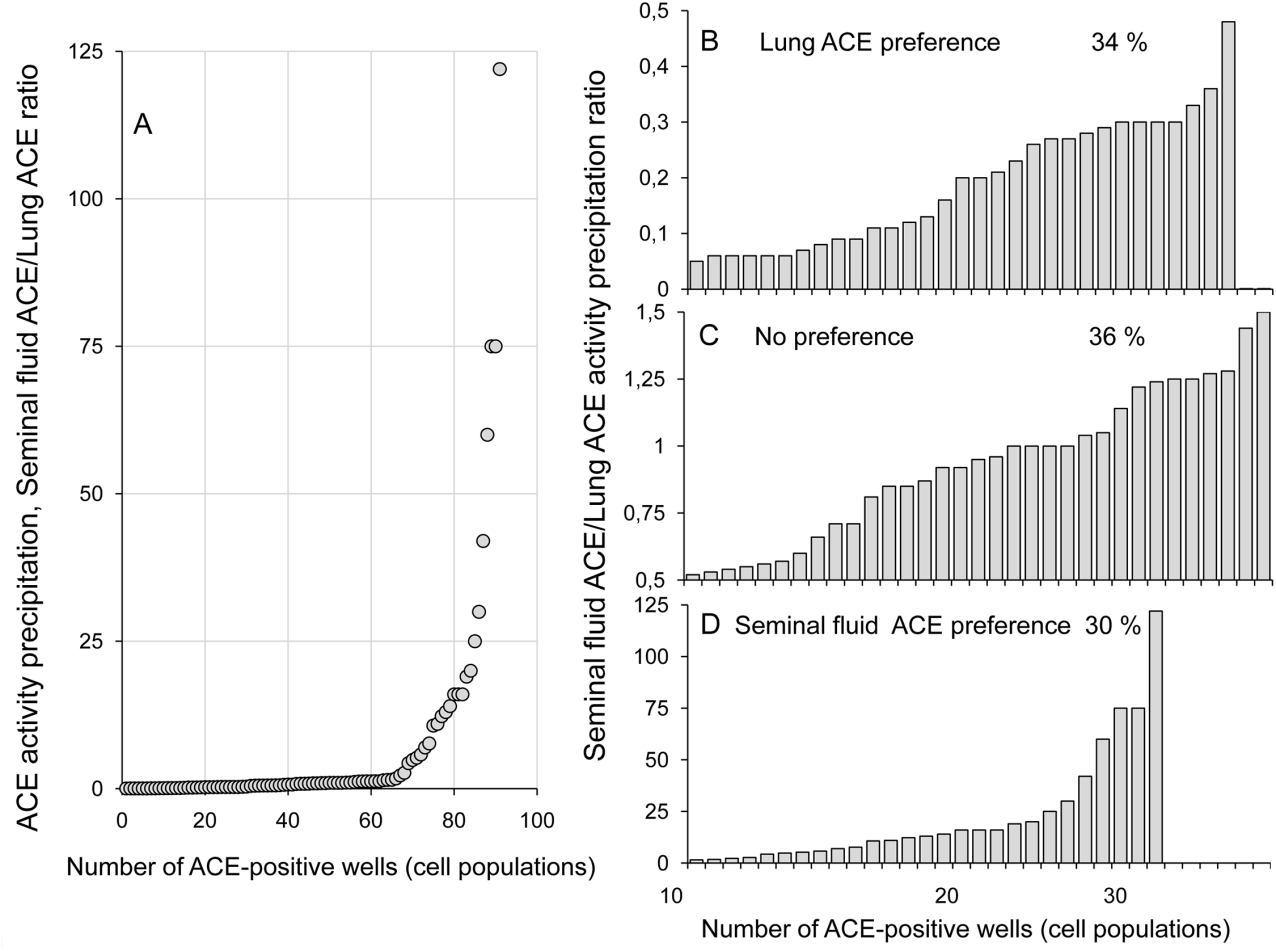
Primary immune response in mice to pure somatic ACE from seminal fluid. Culture fluids from 670 post-fusion cell populations grown in 96-well plates (primary screening) were anylyzed for the presence of antibodies to seminal fluid ACE and lung ACE in parallel in plate precipitation assay (as in Fig 1). Presence of antibodies to seminal fluid and/or lung ACE was detected in 91 wells by precipitated ACE activity and the data are presented as the ratio of ACE activity precipitation from seminal fluid ACE to that from lung ACE (SF/Lung ratio). Discrimination of these two ACEs by antibodies from these positive wells was observed in a wide range (A). Besides expected antibodies, recognizing both ACEs (C) with SF/Lung ratio in the region from 0.5 to 1.5, we identified significant proportions of antibodies which preferentially recognized seminal fluid ACE (B) with SF/Lung ratio more than 1.5, and antibodies which preferentially recognized lung ACE (D) with SF/Lung ratio less than 0.5, correspondingly.

Significant structural differences in ACE from endothelial cells (major source of blood ACE) and ACE from epithelial cells, particularly prostate ACE (demonstrated in this study) and, likely, ovary ACE, may be the base for the development of specific mAbs, which can be used to identify the tissue- or-cell source of increased ACE in blood in different diseases. In order to estimate the possibility of generating monoclonal antibodies which would discriminate between endothelial and epithelial ACEs we attempted immunization of mice with pure somatic ACE from seminal fluid which is the mixture of prostate and epididymal ACEs [15–16]. We used the hybridoma fusion technology to dissect the immune response based on primary screening of post-fusion populations. This would estimate the possibility of generation of tissue ACE-specific antibodies and, eventually, establish antibody-producing hybridoma clones. The results of primary screening of 670 post-fusion populations are shown in Fig 7. The positive response was detected in 91 wells (Fig 7A). The 36% of positive populations reacted rather equally with seminal fluid and lung ACEs (SF/Lung ratio equals to 0.5–1.5, i.e. no significant preference, Fig 7C). The 34% of positive populations demonstrated preference of different degree towards seminal fluid ACE (Fig 7B), while 30% showed preference of different degree to lung ACE (Fig 7D). Therefore, even at the level of polyclonal immune response against seminal fluid ACE it was possible to detect spectrum of antibodies with different preferences to tissue specific ACEs.

## Abbreviations

ACE: angiotensin-converting enzyme
ZPHL: carbobenzoxy-L-phenylalanyl-L-histidyl-L-leucine
HHL: hippuryl-L-histidyl-L-leucine
LMW: low molecular weight

## References

[1] Ehlers MR, Riordan JF (1989) Angiotensin-converting enzyme: new concepts concerning its biological role. Biochemistry 8: 5311–5318. 10.1021/bi00439a001 2476171

[2] Sturrock ED, Anthony CS, Danilov SM (2012) Peptidyl-dipeptidase A/Angiotensin I-converting enzyme In: Rawlings ND; Salvesen G, ed. Handbook of Proteolytic Enzymes, 3rd edn Oxford, Academic Press: 480–494.

[3] Bernstein KE, Ong FS, Blackwell WL, Shah KH, Giani JF, Gonzalez-Villalobos RA, et al (2013) A modern understanding of the traditional and nontraditional biological functions of Angiotensin-converting enzyme. Pharmacol Rev 65: 1–46. 10.1124/pr.112.006809 23257181 PMC3565918

[4] Ching SF, Hayes LW, Slakey LL (1983) Angiotensin-converting enzyme in cultured endothelial cells. Synthesis, degradation and transfer to culture medium. Arteriosclerosis 3: 581–588. 6316884 10.1161/01.atv.3.6.581

[5] Metzger R, Franke FF, Bohle RM, Alhenc-Gelas F, Danilov SM (2011) Heterogeneous distribution of Angiotensin I-converting enzyme (CD143) in the human and rat vascular systems: vessels, organs and species specificity. Microvasc Res. 81: 206–215. 10.1016/j.mvr.2010.12.003 21167844

[6] Hooper NM, Keen J, Pappin DJC, Turner AJ (1987) Pig kidney angiotensin converting enzyme. Purification and characterization of amphipatic and hydrophilic forms of the enzyme establishes C-terminal anchorage to the plasma membrane. Biochem J 247: 85–93. 2825659 10.1042/bj2470085 PMC1148373

[7] Wei L, Alhenc-Gelas F, Soubrier F, Michaud A, Corvol P, Clauser E (1991) Expression and characterization of recombinant human angiotensin I-converting enzyme. Evidence for a C-terminal transmembrane anchor and for a proteolytic processing of the secreted recombinant and plasma enzymes. J Biol Chem 266: 5540–5546. 1848554

[8] Parkin ET, Turner AJ, Hooper NM (2004) Secretase-mediated cell surface shedding of the angiotensin-converting enzyme. Protein Pept Lett 11: 423–432. 15544563 10.2174/0929866043406544

[9] Yokoyama M, Hiwada K, Kokubu T, Takaha M, Takeuchi M (1980) Angiotensin-converting enzyme in human prostate. Clin Chim Acta 100: 253–258. 6153302 10.1016/0009-8981(80)90274-0

[10] Lieberman J, Sastre A (1983) Angiotensin-converting enzyme activity in postmortem human tissues. Lab Invest 48: 711–717. 6304422

[11] Erdös EG, Schulz WW, Gafford JT, Defendini R (1985) Neutral metalloendopeptidase in human male genital tract. Comparison to angiotensin I-converting enzyme. Lab Invest 52: 437–447. 2984462

[12] Vivet F, Callard P, Gamoudi A (1987) Immunolocalization of angiotensin 1 converting enzyme in the human male genital tract by the avidin-biotin-complex method. Histochemistry 86: 499–502. 3034835 10.1007/BF00500623

[13] Nassis L, Frauman AG, Ohishi M, Zhuo J, Casley DJ, Johnston CI et al (2001) Localization of angiotensin-converting enzyme in the human prostate: pathological expression in benign prostatic hyperplasia. J Pathol 195: 571–579. 11745693 10.1002/path.999

[14] Pauls K, Metzger R, Steger K, Klonisch T, Danilov SM, Franke FE (2003) Isoforms of angiotensin I-converting enzyme in the development and differentiation of human testis and epididymis. Andrologia 35: 32–43. 12558527 10.1046/j.1439-0272.2003.00535.x

[15] Hohlbrugger G, Pschorr J, Dahlheim H (1984) Angiotensin I converting enzyme in the ejaculate of fertile and infertile men. Fertil Steril 41: 324–325. 6321247 10.1016/s0015-0282(16)47614-4

[16] Krassnigg F, Niederhauser H, Fink E, Frick J, Schill WB (1989) Angiotensin converting enzyme in human seminal plasma is synthesized by the testis, epididymis and prostate. Int J Androl 12: 22–28. 2541085 10.1111/j.1365-2605.1989.tb01282.x

[17] Nikolaeva MA, Balyasnikova IV, Alexinskaya MA, Metzger R, Franke FE, Albrecht RF 2nd et al (2006) Testicular isoform of angiotensin I-converting enzyme (ACE, CD143) on the surface of human spermatozoa: revelation and quantification using monoclonal antibodies. Am J Reprod Immunol 55: 54–68. 16364013 10.1111/j.1600-0897.2005.00326.x

[18] Fagerberg L, Hallström BM, Oksvold P, Kampf C, Djureinovic D, Odeberg J et al (2014) Analysis of the human tissue-specific expression by genome-wide integration of transcriptomics and antibody-based proteomics. Mol Cell Proteomics 13: 397–406. 10.1074/mcp.M113.035600 24309898 PMC3916642

[19] Danilov SM, Balyasnikova IB, Danilova AS, Naperova IA, Arablinskaya NE, Borisov SE et al (2010) Conformational fingerprinting of the angiotensin-converting enzyme (ACE): Application in sarcoidosis. J Proteome Res 9: 5782–5793. 10.1021/pr100564r 20873814

[20] Kost OA, Bovin NV, Chemodanova EE, Nasonov VV, Ort TA (2000) New feature of angiotensin-converting enzyme: carbohydrate-recognizing domain. J Mol Recognit 13: 360–369. 11114069 10.1002/1099-1352(200011/12)13:6<360::AID-JMR508>3.0.CO;2-K

[21] Bordier C (1981) Phase separation of integral membrane proteins in Triton X-114 solution. J Biol Chem 25: 1604–1607. 6257680

[22] Danilov SM, Balyasnikova IV, Albrecht RF II, Kost OA (2008) Simultaneous determination of ACE activity with two substrates provides information on the status of somatic ACE and allows detection of inhibitors in human blood. J Cardiovasc Pharmacol 52: 90–103. 10.1097/FJC.0b013e31817fd3bc 18645413

[23] Danilov SM, Kalinin S, Chen Z, Vinokour EI, Nesterovitch AB, Schwartz DE et al (2010) Angiotensin I-converting enzyme Gln1069Arg mutation impairs trafficking to the cell surface resulting in selective denaturation of the C-domain. PLoS One 5: e10438 10.1371/journal.pone.0010438 20454656 PMC2862704

[24] Danilov S, Jaspard E, Churakova T, Towbin H, Savoie F, Wei L et al (1994) Structure-function analysis of angiotensin I-converting enzyme using monoclonal antibodies. J Biol Chem 269: 26806–26814. 7523412

[25] Jokubaitis VJ, Sinka L, Driessen R, Whitty G, Haylock DN, Bertoncello I et al (2008) Angiotensin-converting enzyme (CD143) marks hematopoietic stem cells in human embryonic, fetal and adult hematopoietic tissues. Blood 111: 4055–4063. 17993616 10.1182/blood-2007-05-091710

[26] Yu XC, Sturrock ED, Wu Z, Biemann K, Ehlers MRW, Riordan JF (1997) Identification of N-linked glycosylation sites in human testis angiotensin-converting enzyme and expression of an active deglycosylated form. J Biol Chem 272: 3511–3519. 9013598 10.1074/jbc.272.6.3511

[27] Skirgello OE, Balyasnikova IV, Binevski PV, Sun ZL, Baskin II, Palyulin VA et al (2006) Inhibitory antibodies to human angiotensin-converting enzyme: fine epitope mapping and mechanism of action. Biochemistry 45: 4831–4847. 16605251 10.1021/bi052591h

[28] Balyasnikova IV, Skirgello OE, Binevski PV, Nesterovitch AB, Albrecht RF II, Kost OA et al (2007) Monoclonal antibodies 1G12 and 6A12 to the N-domain of human angiotensin-converting enzyme: fine epitope mapping and antibody-based method for revelation and quantification of ACE inhibitors in the human blood. J Proteome Res 6: 1580–1594. 17326675 10.1021/pr060658x

[29] Danilov SM, Watermeyer JM, Balyasnikova IV, Gordon K, Kugaevskaya EV, Elisseeva YE et al (2007) Fine epitope mapping of monoclonal antibody 5F1 reveals anticatalytic activity toward the N domain of human angiotensin-converting enzyme. Biochemistry 46: 9019–9031. 17630779 10.1021/bi700489v

[30] Naperova IA, Balyasnikova IV, Schwartz DE, Watermeyer J, Sturrock ED, Kost OA et al (2008) Mapping of conformational mAb epitopes to the C domain of human angiotensin I-converting enzyme (ACE). J Proteome Res 7: 3396–3411. 10.1021/pr800142w 18576678

[31] Gordon K, Balyasnikova IV, Nesterovitch AB, Schwartz DE, Sturrock ED, Danilov SM (2010) Fine epitope mapping of monoclonal antibodies 9B9 and 3G8, to the N domain of human angiotensin I-converting enzyme (ACE) defines a region involved in regulating ACE dimerization and shedding. Tissue Antigens 75: 136–150. 10.1111/j.1399-0039.2009.01416.x 20003136

[32] Christensen B, Nielsen MS, Haselmann KF, Petersen TE, Sorensen ES (2005). Post-translationally modified residued of native osteoponin are located in clusters: identification of 36 phosphorylation and five O-glycosylation sites and their biological implications. Biochem J 390: 285–292. 15869464 10.1042/BJ20050341 PMC1184582

[33] Brogren H, Sihlbom C, Wallmark K, Lonn M, Deinum J, Karlsson L et al (2008) Heterogeneous glycosylation patterns of human PAI-1 may reveal its cellular origin. Thrombosis Res 122: 271–281. 10.1016/j.thromres.2008.04.008 18508114

[34] West MB, Segu ZM, Feasley CL, Kang P, Klouckova I, Li C et al (2010) Analysis of site-specific glycosylation of renal and hepatic γ-glutamyl transpeptidase from normal human tissue. J Biol Chem 285: 29511–29524. 10.1074/jbc.M110.145938 20622017 PMC2937983

[35] Liddy KA, White MY, Cordwell SJ (2013) Functional decorations: post-translational modifications and heart disease delineated by targeted proteomics. Genome Medicine 5: 20 10.1186/gm424 23445784 PMC3706772

[36] Snider NT, Omary MB (2014). Post-translational modifications of intermediate filament proteins: mechanisms and functions. Nature Reviews Mol Cell Biol 15: 163–177. 10.1038/nrm3753 PMC4079540 24556839

[37] Kohlstedt K, Shoghi F, Muller-Esterl W, Busse R, Fleming I (2002) CK2 Phosphorylates the angiotensin-converting enzyme and regulates its retention in the endothelial cell plasma membrane. Circ Res 91: 749–756. 12386153 10.1161/01.res.0000038114.17939.c8

[38] Soubrier F, Alhenc-Gelas F, Hubert C, Allegrini J, John M, Tregear G et al (1988) Two putative active centers in human angiotensin I-converting enzyme revealed by molecular cloning. Proc Natl Acad Sci USA 85: 9386–9390. 2849100 10.1073/pnas.85.24.9386 PMC282757

[39] Ripka JE, Ryan JW, Valido FA Chung AY, Peterson CM, Urry RL (1993) N-glycosylation of forms of angiotensin converting enzyme from four mammalian species. Biochem Biophys Res Commun 196: 503–508. 8240320 10.1006/bbrc.1993.2278

[40] Lui T, Qian WJ, Gritsenko MA, Camp DG 2nd, Monroe ME, Moore RJ et al (2005) Human plasma N-gycoproteome analysis by immunoaffinity subtraction, hydrazide chemistry, and mass spectroscopy. J Proteome Res 4: 2070–2080 16335952 10.1021/pr0502065 PMC1850943

[41] Lanzillo JJ, Stevens J, Tumas J, Fanburg BL (1983) Spontaneous change of human plasma angiotensin I converting enzyme isoelectric point. Arch Biochem Biophys 227: 434–439. 6320726 10.1016/0003-9861(83)90473-3

[42] Ehlers MRW, Chem Y-NP, Riordan JF (1992) The unique N-terminal sequence of testis angiotensin-converting enzyme is heavily O-glycosylated and unessential fro activity or stability. Biochem Biophys Res Commun 183: 199–205. 1311921 10.1016/0006-291x(92)91628-4

[43] Balyasnikova IV, Sun Z-L, Berestetskaya IV, Albrecht RA II, Sturrock ED, Danilov SM (2005) Monoclonal antibodies 1B3 and 5C8 as probes for monitoring the nativity of C-terminal end of soluble angiotensin-converting enzyme (ACE). Hybridoma 24: 14–26. 15785205 10.1089/hyb.2005.24.14

[44] Danilov SM, Deinum J, Balyasnikova IV, Sun Z-L, Kramers C, Hollak CE et al (2005) Detection of mutated angiotensin I-converting enzyme by serum/plasma analysis using a pair of monoclonal antibodies. Clin Chem 51: 1040–1043. 15914791 10.1373/clinchem.2004.045633

[45] Woodman ZL, Oppong SY, Cook S, Hooper NM, Schwager SL, Brandt WF et al (2000) Shedding of somatic angiotensin-converting enzyme (ACE) is inefficient compared with testis ACE despite cleavage at identical stalk sites. Biochem J 347: 711–718. 10769174 PMC1221007

[46] Fagyas M, Úri K, Siket IM, Daragó A, Boczán J, Bányai E et al (2014) New perspectives in the renin-angiotensin-aldosterone system (RAAS) I: endogenous angiotensin converting enzyme (ACE) inhibition. PLoS One 9: e87843 10.1371/journal.pone.0087843 24691160 PMC3972180

[47] Fagyas M, Úri K, Siket IM, Fülöp GÁ, Csató V, Daragó A et al (2014) New perspectives in the renin-angiotensin-aldosterone system (RAAS) II: Albumin suppresses angiotensin converting enzyme (ACE) activity in human. PLoS One 9: e87844 10.1371/journal.pone.0087844 24691203 PMC3972182

[48] Elzanaty S. Erenpreis J, Becker C (2007) Seminal plasma albumin: origin and relation to the male reproductive parameters. Andrology 39: 60–65. 10.1111/j.1439-0272.2007.00764.x 17430425

[49] Osawa S, Sugo S, Yoshida T, Yamaoka T, Nomura F (2006) An assay for separating and quantifying for bilirubin fractions in untreated human serum using isocratic high-performance liquid chromatography. Clin Chem Acta 366: 146–155. 10.1016/j.cca.2005.09.031 16426596

[50] Beyazit F, Ayhan S, Celik HT, Gungor T (2015) Assessment of serum angiotensin-converting enzyme in patients with epithelial ovarian cancer. Arch Gynecol Obstet 292:415–420. 10.1007/s00404-015-3661-x 25693759

